# Greenhouse Gas Emission Accounting and Management of Low-Carbon Community

**DOI:** 10.1100/2012/613721

**Published:** 2012-12-02

**Authors:** Dan Song, Meirong Su, Jin Yang, Bin Chen

**Affiliations:** State Key Laboratory of Water Environment Simulation, School of Environment, Beijing Normal University, Beijing 100875, China

## Abstract

As the major source of greenhouse gas (GHG) emission, cities have been under tremendous pressure of energy conservation and emission reduction for decades. Community is the main unit of urban housing, public facilities, transportation, and other properties of city's land use. The construction of low-carbon community is an important pathway to realize carbon emission mitigation in the context of rapid urbanization. Therefore, an efficient carbon accounting framework should be proposed for CO_2_ emissions mitigation at a subcity level. Based on life-cycle analysis (LCA), a three-tier accounting framework for the carbon emissions of the community is put forward, including emissions from direct fossil fuel combustion, purchased energy (electricity, heat, and water), and supply chain emissions embodied in the consumption of goods. By compiling a detailed CO_2_ emission inventory, the magnitude of carbon emissions and the mitigation potential in a typical high-quality community in Beijing are quantified within the accounting framework proposed. Results show that emissions from supply chain emissions embodied in the consumption of goods cannot be ignored. Specific suggestions are also provided for the urban decision makers to achieve the optimal resource allocation and further promotion of low-carbon communities.

## 1. Introduction

Global warming has been a hot topic since a few decades ago and became a direct trigger for behavior change for people worldwide [1–35]. As the most impacted region by human activities, cities emit more than 75% of the total greenhouse gas, in which CO_2_ occupies a large proportion [36]. Cities play an important role in global carbon cycle, and most of their impacts are exerted via indirect pathways [37]. With the purpose of the energy resource consumption minimization and greenhouse gas emission reduction, low-carbon cities have attracted increasing attention [38]. As the cell of a city, community is the basic unit in the low-carbon city construction, and its structure and density also play a key role in energy consumption and CO_2_ emission [39, 40]. Low-carbon community provides a platform for individual behavior change [41, 42]. The UK Low-Carbon Transition Plan [43] also makes explicit the major role that households and communities play in building a low-carbon future. A common viewpoint has been reached that low-carbon community will be an efficient way to achieve sustainable development due to its energy utilization, internal structure optimization, and external effects reduction. Obviously, the pursuit of low-carbon community would be extremely essential to retard the global climate change.

In order to estimate the contribution of cities to global climate change, many attempts have been made to quantify the carbon emissions associated with the accounting level in the community. Recently, many organizations have been conducting “low-carbon” projects to estimate the contributions to global climate change. Many protocols were put out to guide organizations to measure GHG emissions [44–46]. These protocols are mainly concentrated on direct emissions and indirect emissions from purchased energy, with less focus on supply chain emissions that occupied a large proportion in a community. For example, direct CO_2_ emissions are found to be generated by direct household energy use, whereas indirect CO_2_ emissions are generated in the industrial sectors producing nonenergy commodities demanded by the households [47]. Pachauri and Spreng applied the IO models into the calculation of direct and indirect energy consumption of households in India based on the 115-sector classification input-output tables [48]. Lu et al. quantified the direct and indirect household emissions of CO_2_ in China with the help of input-output life-cycle assessment (IO-LCA) combined with 8 categories of household expenditure [49]. A calculation framework for whole life-circle carbon budget in residential area was presented based on building system, social system, and green space system, showing that the ratio of carbon source to carbon sink is 29 : 1 and that of society source to building source is 4.6 : 1 [50]. It can be seen that there is serious imbalance between carbon sink and carbon source in this residential area, and the society source is a key factor for carbon budget balance.

Moreover, Matthews et al. classified the variety scopes of carbon footprint into 3 tiers, including direct emissions, emissions from purchased energy, and supply chain emissions [51]. In their study, two case studies of book publishers and power generation were conducted, which illustrated that the first 2-tier emissions accounted for only a small part while a large portion is constituted by emissions embodied in the supply chain. The Scope 3 footprints of US economic sectors using a modified form of the 2002 US benchmark Economic Input-Output Life Cycle Assessment (EIO-LCA) model was developed to categorize upstream emission sources [52]. Larsen and Hertwich developed a greenhouse gas emissions inventory related to the provision of municipal services in the city of Trondheim, Norway, indicating that approximately 93% of the total carbon footprint of municipal services is indirect emissions [53]. The authors also established CO_2_ inventories focused on the supply chain emissions of CO_2_ emissions from each sector, for example, agriculture, industry, transportation, and tertiary industry, and identified the sectors that contribute the most to climate change [54]. 

As can be seen, the previous studies on 3-tier accounting are mainly concentrated on industry sectors, with less focus on community-level CO_2_ emissions. A special focus should be transferred to identify Scope 3 categories that are relevant and incorporated into the footprint analysis. Thus, further characterization of the total supply chain emissions in community is necessary in order to achieve a better strategy for carbon emission mitigation. Approaches based on life cycle assessment (LCA) methods are available to estimate the embodied CO_2_ in the consumption of goods, which provides a framework for analysis of the potential environmental impacts embodied throughout the lifetime of goods [55, 56]. There are two common types of LCA models, that is, process-based LCA and EIO-LCA, varying according to differences in system scope and analysis with its own processes and characteristics [57]. Economic IO models were first developed by Leontief in 1936 to aid manufacturing planning [58]. Compared to the process-based LCA, EIO-LCA addresses some of the drawbacks of process-based LCA model and greatly expands the system scope to include the entire economy of a region, which can assess the energy consumption and environmental impacts of goods from a nationwide perspective based on economic input-output matrix. 

The aim of this paper is to propose an efficient three-tier carbon emission accounting framework for community. Taking a typical high-quality community in Beijing as case study, this study also intends to quantify the magnitude of carbon emissions and the mitigation potential using the method of LCA in combination with a detailed CO_2_ emission inventory, including emissions from direct fossil fuel combustion, emissions from purchased energy (mainly contains electricity, water, and heat), and supply chain emissions embodied in the consumption of goods. Some suggestions about the realization of optimized resource allocation and further promotion of such communities are also given for the decision makers.

## 2. Methodology

We develop estimation equations for three tiers of carbon footprint of the community based on the scope initially developed by Matthews et al. [51].

Tier 1 includes direct emissions from household fossil fuel combustion and vehicles, including emissions from natural gas, gasoline, diesel oil, and jet kerosene. This is similar to the “consumer perspective” used for emissions inventories [59].

Tier 2 is based on Tier 1, in addition to indirect emissions from purchased energy (mainly contains electricity, water, and heat) for a community.

Tier 3 includes the total supply chain emissions embodied in the consumption of goods and activities. The accounting model and boundaries used for estimating all purchases and activities aspects in a supply chain by any sector of a community are based on EIO-LCA, which are consistent with the data structure described in Section 3.2.

The decomposition analysis is carried out in two steps. Firstly, Tier 1 and Tier 2 CO_2_ emissions from household energy use are analyzed using a simple energy emission model. Secondly, Tier 3 CO_2_ emissions are analyzed using an extended LCA model that also incorporates energy and emission matrices.

In terms of spatial system boundary, the total CO_2_ emissions are derived from emissions from household and public area. Thus the total CO_2_ emissions calculated in 3 tiers can be defined as

(1) E=Eh+Ep,Eh=Eh1+Eh2+Eh3+Eh4+Eh5+Eh6+Eh7+Eh8+Eh9,Ep=Ep1+Ep2,

where *E* is the total CO_2_ emissions from community; *E*_*h*_ refers to all the three-tiers CO_2_ emissions from household that consists of CO_2_ emissions from direct energy consumption (*E*_*h*1_), indirect energy and water consumption (*E*_*h*2_), transport and community (*E*_*h*3_), food (*E*_*h*4_), clothing and footwear (*E*_*h*5_), household appliances and services (*E*_*h*6_), healthcare (*E*_*h*7_), education and recreation (*E*_*h*8_), and from buildings (*E*_*h*9_); *E*_*p*_ refers to CO_2_ emissions from the public area of a community that consisted of CO_2_ emissions from electricity consumption (*E*_*p*1_) and from water consumption (*E*_*p*2_).

## 3. Case Study

### 3.1. Study Area

As Beijing is in its fast process of urbanization, community construction turns into a key element of the city renovation. This paper selects a typical high-quality community in Beijing as the case study. The community covers an area of 8.2 × 10^3^ m^2^ with a construction area of 3.0 × 10^5^ m^2^ and a living area of 9.0 × 10^4^ m^2^. The community has 1630 households and a permanent population of 3100, with a green space of more than 2500 m^2^ and a greening rate of 30%. The community has carried out the garbage classification since 2004. So far, the capacity of the kitchen waste disposal equipment that came into use has reached 20 kg per day. The power consumption is 2.24 × 10^5^ kWh per month, and water consumption is about 1.63 × 10^4^ m^3^ per month.

### 3.2. Data Analysis

CO_2_ emission factors of primary energy are based on the CO_2_ content of the fuels and the type of energy, which are elaborated in IPCC [60]. CO_2_ emissions factors of electricity are based on coal factors but corrected by standard coal consumption of power supply (standard coal consumption 356 g/kWh, the average value in China [61]). CO_2_ emissions factors for renewable energy are considered to be zero. The CO_2_ emissions factors of energy are shown in Table 1. Other CO_2_ emission factors of consumption goods can be referred to the embodied greenhouse gas emission database [62].

In this study, direct CO_2_ emissions from the consumption of electricity and heating are not considered. The energy inputs for the production of electricity and district heating are estimated as the final consumption of energy production; that is, all emissions caused by energy production are specified for each of the fuel inputs [56].

The consumption data are developed based on the survey carried out in the community. Based on the previous studies engaged to classify the sectoral composition of consumption [48, 49, 63], we aggregate the community consumption in the database into the same expenditure framework, of which 8 emission categories include food, clothing and footwear, household appliances and services, health care, transport and communication, education and recreation, building, and miscellaneous goods, as listed in Table 2.

## 4. Results

### 4.1. Comparison of Tier 1, Tier 2, and Tier 3 CO_2_ Emissions

The results show that the first 2 tiers defined by the current most carbon footprint protocols only occupy a small fraction of the total supply chain (Tier 3). Direct emissions from the community are only 1.58% of the total emissions, and on average only 11.46% of Tier3 are captured by Tier 2. The major carbon source is the total supply chain emissions embodied in the consumption of goods and activities, which is called Tier 3. Thus reduction emphasis should be put on Tier 3. From this aspect we can see that a large quantity of CO_2_ emissions may be underestimated according to the current estimation protocols. 

### 4.2. CO_2_ Emissions Structure

**Figure 1:**
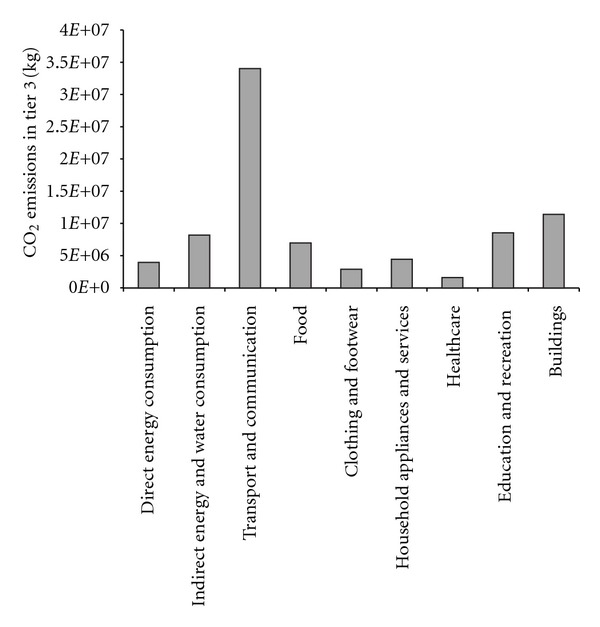
Total CO_2_ emissions in Tier 3.

For the total CO_2_ emissions, which are defined as Tier 3, the top 3 emission items are transport and communication (41.36%), buildings (14.11%), and education and recreation (10.41%), as shown in Figure 1. Income is an important factor for CO_2_ emission. In a typical high-quality community of Beijing, residents enjoy a high-standard life and prefer more convenient and faster communication tools. Thus more private cars and advanced communication tools are needed, which add to the total emissions.

The buildings consume a large amount of materials, equipment, energy, and manpower at the stages of construction, fitment, outdoor facility construction, transportation, operation, waste treatment, property management, demolition, and disposal [64]. Due to a lack of data, only the main material consumption is considered in this study. Although this part occupies 14.11% of the total CO_2_ emission, it is still smaller than the real value.

 Energy consumption tends to increase along with income rise, which is confirmed by numerous studies [65, 66]. Thus, the main CO_2_ emissions are from goods purchasing. The expenditure of health care is the smallest, which is mainly due to the age structure present in this community.

### 4.3. Comparison with Nanjing Community

There is a previous study on the typical community of Nanjing-Zhujiang Road Community (termed as Site A) [67]. Per capita CO_2_ emissions of Site A from electricity, natural gas, and petrol consumptions are 1144.5 kg, 48.7 kg, and 540.1 kg, while in our case are 974.19 kg, 374.19 kg, and 893.55 kg, respectively. CO_2_ emission from electricity of Beijing case is 14.88% lower than that of Site A. The younger residents in Beijing community have a better sense of energy conservation and usually prefer energy saving appliances. The CO_2_ emission from natural gas of Beijing case is nearly seven times higher than that of Site A because space heating in northern China contributes the most while people do not have heating services in southern China like Nanjing. Meanwhile, the CO_2_ emissions from petrol consumption of Beijing case are 65.44% higher than that of Site A due to longer distance between working place and home in Beijing compared to Nanjing. Particularly, our case considers the total emissions embodied in the supply chain, which is often significantly underestimated by the previous studies.

## 5. Conclusions

In this paper, a new carbon accounting framework, that is, three-tier accounting method, was established to estimate the total embodied CO_2_ emissions of urban community. The carbon emissions and the mitigation potential were quantified according to the proposed accounting framework. From the results we can obtain that in the concerned community only 11.46% of Tier 3 are captured by Tier 2. The major carbon source is the total supply chain emissions embodied in the consumption of goods and activities. The results also indicated that for the total CO_2_ emissions, the top 3 emission items are transport and communication (41.36%), buildings (14.11%), and education and recreation (10.41%).

As can be seen, the mitigation emphases should be placed on Tier 3. Two major suggestions are thereby provided to realize the optimal resource allocation and further promotion for such communities. One is that we should strengthen the promotion of energy-efficient or green building and pay more attention to the renewable energy appliances such as solar energy water heater. The architectural of the houses should also be improved to reduce energy consumption of lightning and space heating. On the other hand, due to public transportation, the reconstruction of the urban public transportation is needed to reduce CO_2_ emissions caused by the huge growth of private car ownership. 

## Figures and Tables

**Table 1:** The CO_2_ emissions factors of conventional energy.

	Coal (KgCO_2_/GJ)	Natural gas (KgCO_2_/GJ)	Electricity (KgCO_2_/kWh)	Gasoline (KgCO_2_/GJ)
CO_2_ emission coefficients	110.08	56.10	1.15	69.30

**Table 2:** Consumption categories of the community.

No.	Items	Contents
1	Food	Miscellaneous food products, beverages, and tobacco products.
2	Clothing and footwear	Miscellaneous textile products, leather footwear.
3	Household appliances and services	Electrical appliances (television, computer, and other electrical machinery). Furniture and fixtures, wood products, and kitchen appliances.
4	Healthcare	Cosmetics, medical and health services, and other services.
5	Transport and communication	Communication equipments, ships and boats, railway, motor vehicles, bicycles, other transportation ways, and other transport services.
6	Education and recreation	Paper, paper products and newspapers, printing publishing and similar activities, and education and research.
7	Buildings	Residence and public buildings.
8	Misc goods and service	Trade, banking, insurance, and so forth.
9	Direct energy consumption	Natural gas, gasoline, diesel oil, and jet kerosene.
10	Indirect energy and water consumption	Electricity, heat, and water.
